# Carbon Dots as Nano‐Photocatalysts: A Green Tool for Indole and Heteroarenes Alkylation

**DOI:** 10.1002/chem.202502043

**Published:** 2025-09-16

**Authors:** Jules Sargueil, Marta Santoni, Simone Cichella, Antonio Di Sabato, Vyali Georgian Moldoveanu, Elisa Sturabotti, Beatrice Simonis, Mauro Giustini, Luisa Maria Migneco, Francesca Leonelli, Fabrizio Vetica

**Affiliations:** ^1^ Department of Chemistry Sapienza University of Rome Piazza Aldo Moro 5 Rome 00185 Italy; ^2^ PPSM Université Paris‐Saclay CNRS ENS Paris‐Saclay Gif‐Sur‐Yvette 91190 France; ^3^ Center for Cooperative Research in Biomaterials (CIC biomaGUNE) Paseo de Miramón 194 Donostia‐San Sebastián 20014 Spain; ^4^ Sede secondaria di Roma – Meccanismi di Reazione c/o Dipartimento di Chimica Institute for Biological Systems (ISB) Italian National Research Council (CNR) Piazzale Aldo Moro 5 Rome 00185 Italy

**Keywords:** carbon dots, indoles functionalization, heteroarenes, photocatalysis, recyclable catalyst, sustainable chemistry

## Abstract

Taking into account the hurdles of climate change and environmental pollution, through the years, the development of sustainable protocols in catalysis stands as a cornerstone to answer those issues. Carbon dots (CDs) have recently proven to be efficient in various types of catalysis, including photocatalytic reactions, while being a green and economic alternative for metal catalysts. In this paper, we present a new catalytic system for the photoredox functionalization of indoles and heteroarenes derivatives based on CDs as nano‐photocatalysts. This reaction, based on the cooperation between our catalyst and 2,6‐lutidine, shows the ability to functionalize these compounds up to 88%, it is effective on 1 mmol scale, and shows a recyclability potential at least to 5 cycles. This system also demonstrated the capacity of obtaining rare types on indoles, having two electron withdrawing groups (EWG), both on the carbon 2 (C2) and carbon 3 (C3). Furthermore, careful studies led to the partial resolution of the mechanism, and to explanations on the regioselectivity of the reaction, which has been studied through the nature of both reagents and substrates, but also through the light's wavelength used in the reaction.

## Graphical abstract

A nano‐photocatalytic method for the functionalization of indoles and heteroarenes is reported. The reaction is promoted by the photocatalytic activity of carbon nanodots, which was thoroughly investigated by mechanistic studies to determine the reaction mechanism. The developed procedure allowed for the preparation of alkylated heteroarenes with good yields (up to 88%), with good functional group tolerance, with the possibility to recycle the nanocatalysts up to 5 times without hindering the reaction outcomes.

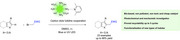

## 1 Introduction

**Scheme 1:**
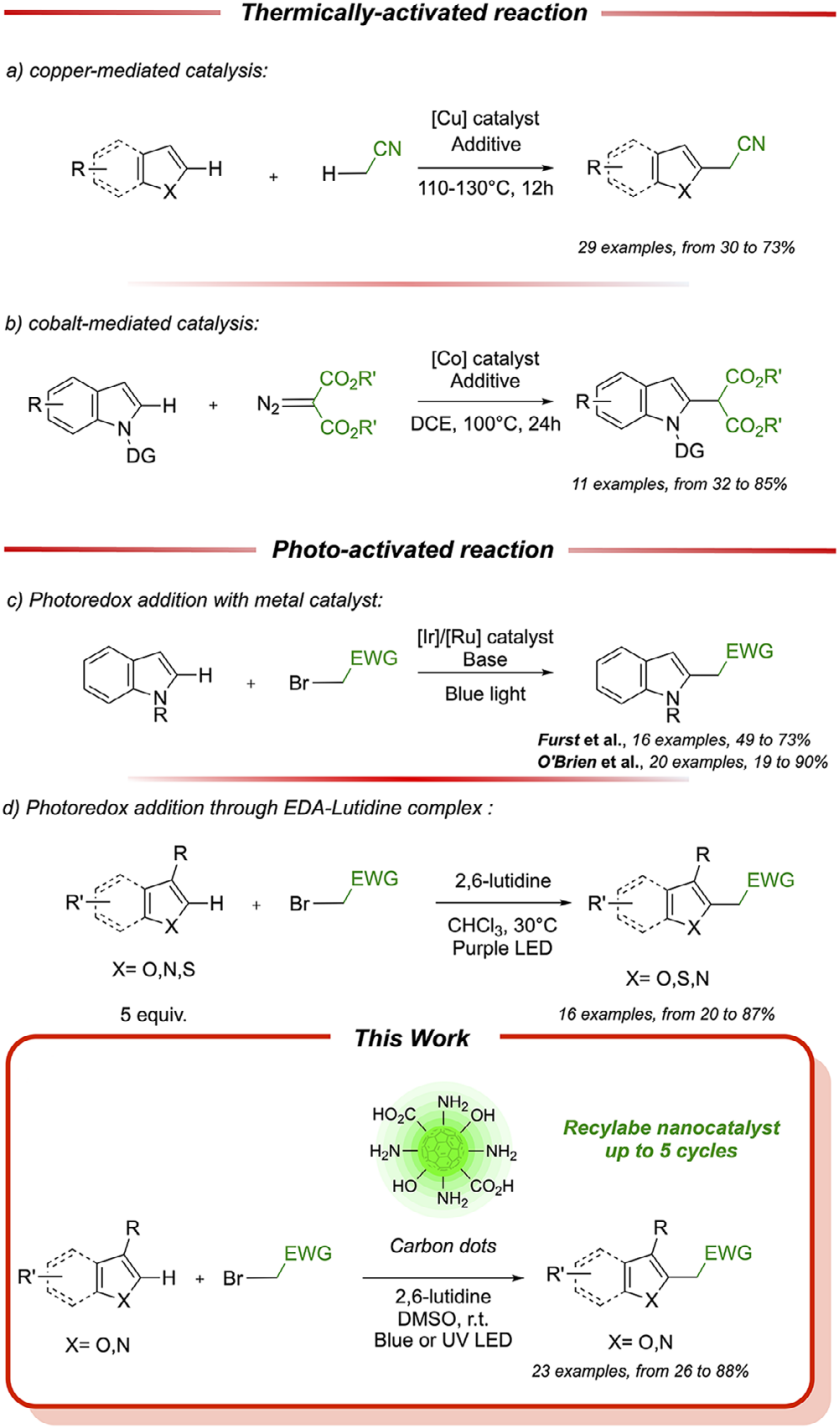
Synthesis of C2 substituted indoles with EWG. Thermically‐activated reaction (a) copper‐mediated and b)cobalt mediated catalysis); photo‐activated reaction (c) metal catalysis and d) EDA‐lutidine complex); this work.

Molecules containing an indole core are nowadays of great interest in the world of chemical industry. As they usually show biological activity,^[^ ^1^ ^]^ and in the pharmaceutical^[^ ^2^ ^]^ and agrochemical fields.^[^ ^3^ ^]^ Activity and mechanism of indole‐based anticancer drugs are really well depicted in literature.^[^ ^4^ ^]^ Currently, a deeper understanding of correlation between active indole structure and reactivity is highlighted,^[^ ^5^ ^]^ thus fostering the search of new derivatives suitable for curing various diseases. Even if C2 functionalization is well precedented,^[^ ^6^ ^]^ compared to C4 to C7 substitutions,^[^ ^7^ ^]^ derivatizations on C3 are still more favorable than the C2 ones.^[^ ^8^ ^]^ Furthermore, most of C2 functionalizations are not reported with electron withdrawing groups (EWG). The EWG is always used as a directing group,^[^ ^9^ ^]^ which can be destroyed or alternated for the sake of the reaction.^[^ ^10^ ^]^ Alternatively, the reaction can proceed through the thermodynamically favored formation of a tricyclic indole intermediate, which may tolerate a C3 EWG,^[^ ^11^ ^]^ as the EWG can also serve as a directing group.^[^ ^12^ ^]^ However, as previously mentioned, these reactions are generally disfavored due to electronic deactivation at the C2 position, and substrates bearing C3 EWGs are often reported as unreactive for C2 functionalization.^[^ ^13^ ^]^ Nevertheless, some specific cases, typically involving 3‐chloro‐substituted indoles, have been described.^[^ ^14^ ^]^ This type of reaction is described using thermically activated metal complexes, such as copper catalysts for acetonitrile addition (Scheme 1a),^[^ ^15^ ^]^ or cobalt catalysts for malonates moieties (Scheme 1b).^[^ ^16^ ^]^ Following more sustainable pathways, photo‐catalytic reactions mediated by noble‐metal catalysts emerged, proceeding through the generation of radical species (Scheme 1c),^[^ ^17^ ^]^ Nonetheless, these types of catalysts are still based on noble metals species, and as previously stated, are still not capable to accomplish C2 functionalization for C3‐EWG‐substituted indoles. Recently, a study from Trapp research group highlights a metal‐free single step reaction capable of affording C2‐diethylmalonate or C2‐acetonitrile substituted heteroarenes, such as indoles, thanks to the formation of an Electron Donor‐Acceptor (EDA) complexes with 2,6‐lutidine (Scheme 1d).^[^ ^18^ ^]^

Due to this state of the art and our interest in the synthesis of potentially biologically relevant heterocyclic scaffolds,^[^ ^19^ ^]^ we decided to implement a new photocatalytic system for versatile EWG functionalization on indoles and other heteroarenes. Metal‐free photocatalysts are widely sought after in order to overcome the shortcomings of transition metal complexes, namely toxicity, high costs and environmental unsustainability. In this regard, a class of biocompatible and low‐cost carbon‐based nanoparticles, known as carbon dots (CDs), is emerging as a possible substitute for metal‐based photocatalysts.^[^ ^20^ ^]^ Thanks to their structure and their superficial functionalization, both easily modulated by simple adjustments in the conditions of their one‐step synthesis, CDs are now being studied as very useful material for different type of application, including direct environmental,^[^ ^21^ ^]^ medicinal,^[^ ^22^ ^]^ sensing,^[^ ^23^ ^]^ and catalysis purposes.^[^ ^24^ ^]^ By exploiting these new environmentally friendly, nontoxic, and inexpensive materials, researchers have sought to develop the library of photo‐catalyzed reactions using CDs, as much as possible, avoiding the use of rare and toxic metals.^[^ ^25^ ^]^ CDs have been previously employed successfully in perfluoroalkyaltions,^[^ ^25d^ ^]^ aldol condensations,^[^ ^26^ ^]^ ring opening,^[^ ^27^ ^]^ cycloadditions,^[^ ^25f^ ^]^ C─C coupling reactions,^[^ ^28^ ^]^ or recently in indole functionalization on the C3 position.^[^ ^29^ ^]^ In our previous work we were also able to successfully employ CDs as photocatalysts in an enantioselective α‐alkylation of aldehydes with EWG groups.^[^ ^30^ ^]^ On the basis of the results obtained in our previous study, here we focus on another unfavored alkylation, the insertion of electron‐poor groups on the C2 position of indoles. As a reaction of pharmaceutical relevance, the use of transition metal complexes is undesirable due to not only the aforementioned issues but also the necessity of additional treatments, to reduce the residual toxic catalysts from active pharmaceuticals.^[^ ^25^, ^31^ ^]^ Employing CDs as green, nontoxic, cheap, and easy‐to‐make nanoparticles would allow to resolve these issues.

## 2 Results and Discussion

In our previously reported work, we studied the application of different CDs in the photochemical activation of haloalkanes by generating electrophilic radicals for the α‐functionalization of aldehydes.^[^ ^30^ ^]^ With these results, we envisioned a subsequent work to employ the photochemically generated alkyl radicals to alkylate the C2 position of indoles and other heteroarenes.

Initially, we decided to proceed with a further photochemical characterization of our sets of nanoparticles. Specifically, we decided to first estimate their excited state redox potentials.

**Figure 1:**
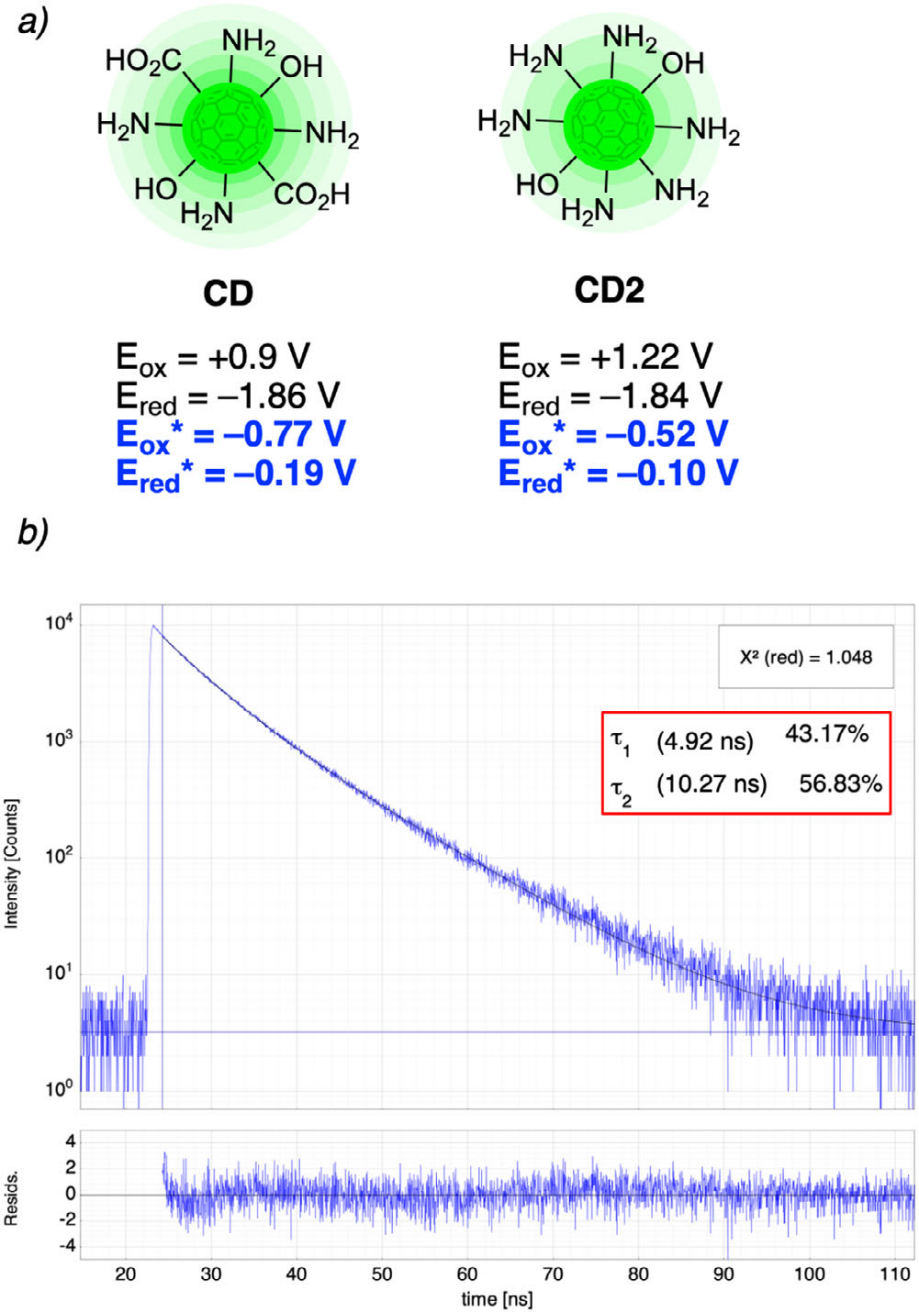
a) Reduction and oxidation potentials both for the ground^[^ ^30^ ^]^ and excited states of the designed nanocatalysts. Excited state potentials were determined by Rehm–Weller equation (*vide infra*); b) fluorescence lifetime decay and fitting curve of **CD**; the inset shows the values of the two fluorescence lifetimes with the relevant weights.

The ground state oxidation and reduction potentials were previously determined by cyclic voltammetry experiments (Figure 1a). According to the Rehm–Weller theory, it is possible to estimate the excited state redox potentials, based on the ground state potentials and the energy difference (E_0,0_) between the lowest vibrational level of the ground state S_0_ and the corresponding excited state S_n_ or T_n_ (Equation 1). E_0,0_ can be roughly estimated from the position of the long wavelength tail (*λ*_tail_) of the absorption spectrum (see Supporting Information for details).^[^ ^32^ ^]^ On this basis, we were able to calculate the excited states redox potentials of both sets of CDs (Figure 1a).

$$\begin{array}{ccc} E_{\mathrm{ox}}^{*} & = & E_{\mathrm{ox}}-E_{0,0}\\ E_{red}^{*} & = & E_{red}+E_{0,0} \end{array}$$

**Equation 1**. Rehm–Weller equation for the estimation of the excited states redox potentials. *E*_ox_/*E*_red_ are the redox potentials of the ground state, measured via cyclic voltammetry; *E*_0,0_ is the energy gap between the zeroth vibrational levels of the ground and excited states.

Then, we decided to measure their fluorescence lifetimes, to subsequently calculate the quenching constants for our photoredox processes. For both sets of nanoparticles the measured fluorescence lifetimes revealed a biexponential fluorescence decay for **CD** (average *τ* = 7.96 ns) and a three‐exponential nature for **CD2** (average *τ* = 6.50 ns) (Figure 1b and Supporting Information, page 7).

**Scheme 2:**
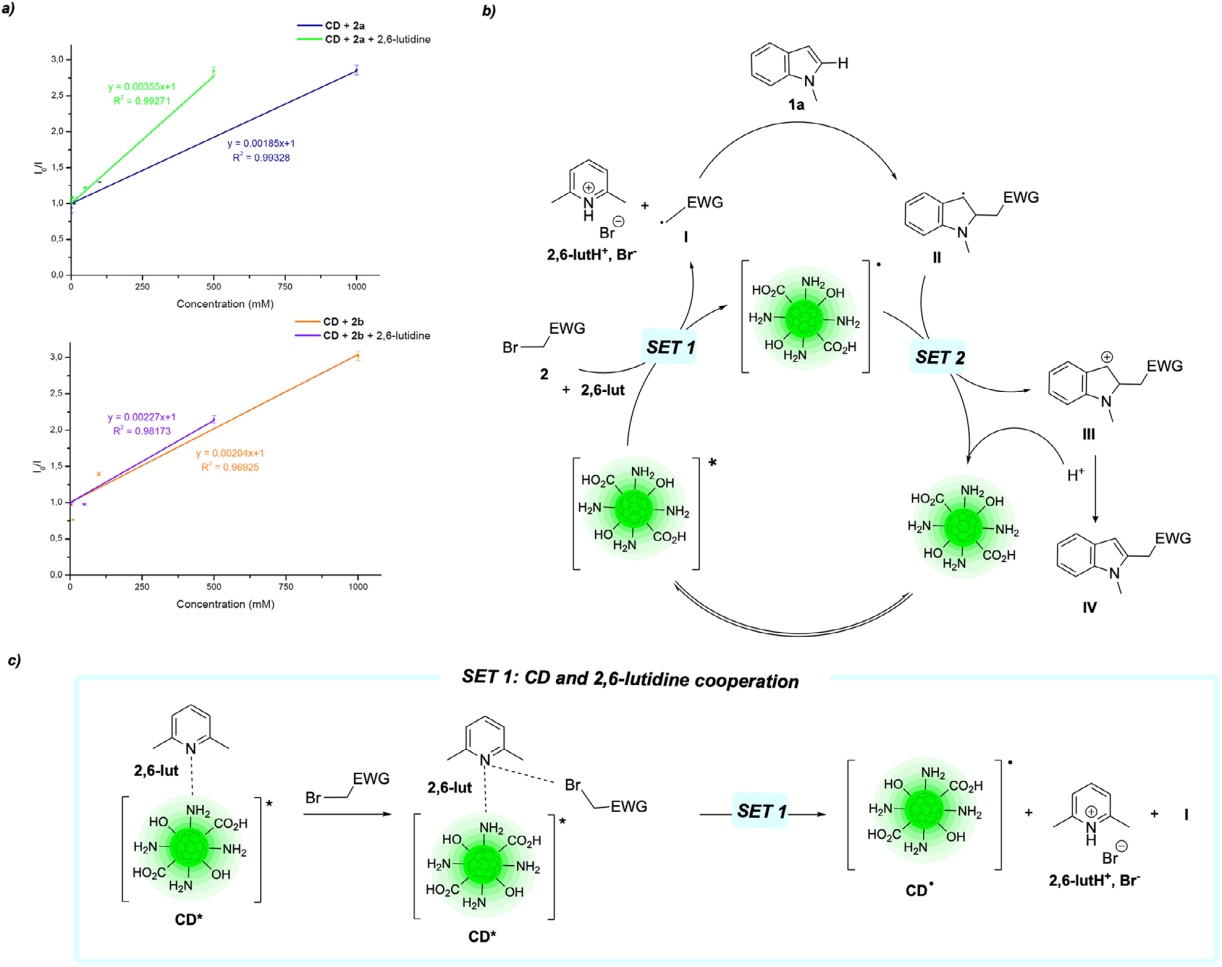
a) Stern–Volmer plots for diethyl bromomalonate (**2a**) and bromoacetonitrile (**2b**) in solution with **CD** and with or without 2,6‐lutidine. b) Proposed catalytic cycle for the photoredox functionalization of indoles thanks to **CD**. c) Proposed explanation for the observed cooperation between **CD** and 2,6‐lutidine, occurring during SET1.

In our previous work, we ascertained the necessity of the presence of 2,6‐lutidine to generate the alkyl radical. In fact, the addition of 2,6‐lutidine to a solution of CDs increased the current intensity in the voltammograms, without shifting the value of the ground state potential.^[^ ^30^ ^]^ Nevertheless, the exact role of lutidine in the reduction of the alkyl halide was not investigated in detail. Once established the photochemical behavior of our CDs, we decided to further investigate this phenomenon through fluorescence quenching experiments. Both the quenching of the photocatalyst in the presence of the haloalkanes and of a mixture of the haloalkanes and 2,6‐lutidine have been performed and the relevant Stern–Volmer analysis carried on. Since the first set of CDs (**CD**) gave better photocatalytic performances, thanks to its high quantum yield and strong fluorescence emission, we selected it as model photocatalyst for the Stern–Volmer experiments (raw quenching data are included in the Supporting Information, page S4). From the fits (Scheme 2a), it is clear that 2,6‐lutidine plays a role in the quenching of CDs, in particular with diethyl bromomalonate, as it can be seen by the increase in the slope. The Stern–Volmer constant (*K*_SV_) increased from 1.9 to 3.6 M^−1^. Considering that the quenching constant (*k*_q_) is dependent on both the *K*_SV_ and the lifetime of the fluorescence emissive excited state (*τ*_0_) (Equation 2), which is the same in both experiments, a more efficient quenching in the presence of lutidine has been demonstrated, thus further confirming the previous observation in which lutidine is enhancing the electron transfer process during the redox process.

$$K_{SV}=k_{q}\;\tau_{0}$$

**Equation 2**. Stern–Volmer constant equation where *k*_q_ is the bimolecular quenching constant and *τ*_0_ is the fluorescence lifetime.

Such clear difference in the slope of the bromoacetonitrile was not measured, leading to the conclusion that between these two substrates, diethyl bromomalonate in the presence of 2,6‐lutidine turns out to be a better quencher of **CD**. This was further confirmed by calculating the bimolecular quenching constants (*k*_q_) (see Supporting Information).

**Scheme 3:**
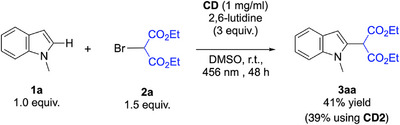
Initial test reaction between **1a** and **2a**.

Once demonstrated the role of lutidine in the generation of alkyl radicals, we decided to harvest this method to functionalize indoles. We selected *N*‐methylindole (**1a**) and 2‐bromomalonate (**2a**) as model substrates for an initial test reaction in the presence of 2,6‐lutidine (Scheme 3). With our delight, the envisioned alkylated product on the C2 position was obtained with moderate yield of 41%.

Prompted by the initial promising results and considering the Stern–Volmer plots, we were able to propose a mechanism in two parallel parts on the indole **1a**. In the first place, a classic photo‐catalysis cycle is proposed in Scheme 2b. On the latter, the **CD**, excited by blue or purple LED, is able to reduce haloalkane **2** to radical **I** through a single electron transfer (SET1). This radical then reacts with the indole **1a** to generate the electron rich molecule **II**, which, through a second SET (SET2), forms the cation **III** and lets the **CD** return to its initial state. Eventually, **III** changes to **IV** by the favorable loss of a proton, captured by the negatively charge **CD**, leading to the resurgence of the indole aromaticity. Even if the efficiency of **CD** alone is shown in Scheme 2a, this catalytic cycle does still not explain the reason why 2,6‐lutidine seems to play a significant role in our reaction. Supported by the Stern–Volmer plots presented above and literature data in which a base could enhance SET mechanism by deprotonating the generated radical cation of the photocatalyst during its generation,^[^ ^33^ ^]^ we postulate a **CD**‐2,6‐lutidine cooperation during SET1. This is complemented by the heteroarenes base electronic properties and by the interaction of the base with both reagent **2** and **CD** (Scheme 2c). This link, obtained by the presence of this specific base, helps to reduce **2** to **I** and to generate the radical **CD**. This was furthermore confirmed by the results of the base screening through the reaction optimization.

To further confirm the postulated mechanism, we decided to calculate the quantum yield (*Φ*) of the reaction via actinometry, which was 0.0011 for the model substrate **3aa** obtained under optimized reaction conditions (see Supporting Information). Being the quantum yield lower than **1**, we can confidently exclude any radical chain propagation pathways, further validating our postulated mechanism.

In order to gain a deeper understanding of the mechanism, different bases were used to prove the cooperation between 2,6‐lutidine and **CD**. When employing other bases, including both mineral bases (Table 1, entry 2) and alkyl amines (entry 3), no product formation was observed, unlike the low yield obtained when we didn't employ any base at all (entry 9). Only an aromatic base structurally similar to lutidine, symmetric collidine (entry 4), yielded the desired product.

**Table 1:** Reaction optimization.

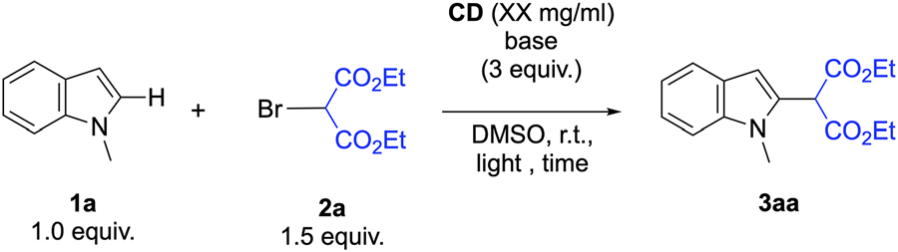				
Entry	Base	CD [mg/mL]	Time, light	Yield[%]^[^ ^a^ ^]^
**1**	2,6‐lutidine	1	65 h, 456 nm	46
**2**	CsHCO_3_	1	48 h, 456 nm	n.r. ^[^ ^b^ ^]^
**3**	DBU	1	48 h, 456 nm	n.r. ^[^ ^b^ ^]^
**4**	2,4,6 collidine	1	48 h, 456 nm	28
**5**	2,6‐lutidine	1	65 h, 390 nm	52
**6**	2,6‐lutidine	3	65 h, 390 nm	70
**7**	2,6‐lutidine	4	65 h, 390 nm	56
**8**	2,6‐lutidine	0	65 h, 390 nm	37
**9**	No Base	3	65 h, 390 nm	19
**10**	2,6‐lutidine	3	65 h, Dark	traces

^[a]^ Yield determined via ^1^H NMR analysis using the internal standard method.

^[b]^ n.r. = no reactions.

Then, the quantity of **CD** employed in the reaction was optimized, obtaining the highest yield by using 3 mg/mL of the latter (see entries 5, 6, and 7). More energetic beams were also required to irradiate the reaction mixture (entries 4 and 5). This is probably due to a higher concentration of generated radicals, which presumably matches the kinetic of the indole functionalization reaction. Entries 8 and 9 of Table 1 highlight the necessity of both CD and 2,6‐lutidine to ensure a satisfactory reactivity, as separate experiments without either of the two led to yields lower than entry 5, even considering their combination, further confirming the synergistic action of the CD and the aromatic base. Finally, the photocatalytic process was proven by a last control experiment in the dark, leading to the obtainment of only traces of the product **3aa** (entry 10). These parameters and others were also investigated with bromo acetonitrile **2b**, such as solvent (further information are provided in the Supporting Information).

**Scheme 4:**
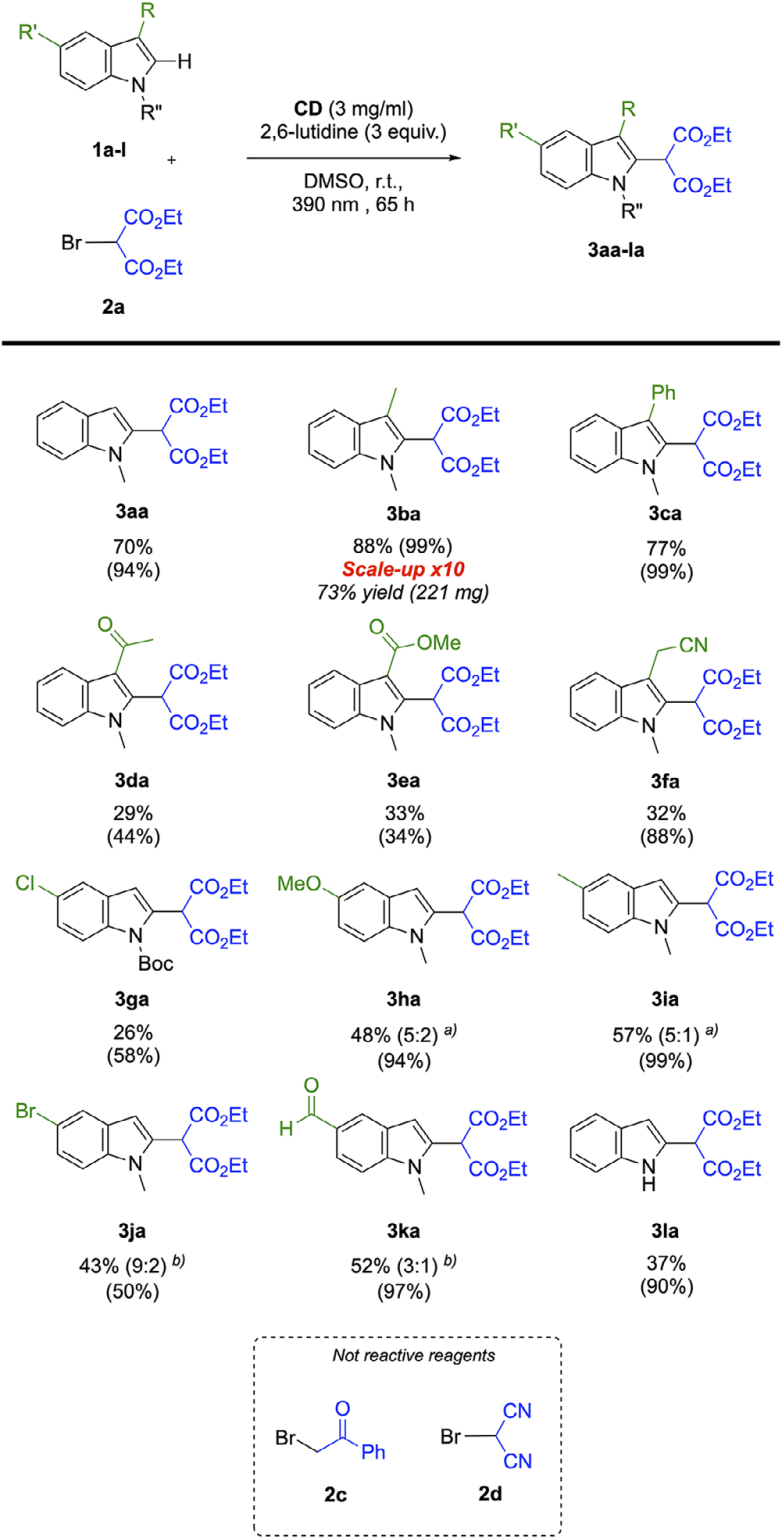
Substrate scope for the photoreaction with bromomalonates **2a** and indole derivatives. Values in brackets represent the conversion values of the indole substrates, calculated via ^1^H NMR of the crude. Unless otherwise noted the reaction conditions were: **1** (0.1 mmol), **2** (0.15 mmol), 2,6‐lutidine (0.3 mmol), **CD** (3 mg/mL) were dissolved in 1 mL of DMSO, degassed with Ar for 20 minutes and left stirring under irradiation for the indicated time. a) regioisomeric ratio refers to C2:C4 regioisomers; b) regioisomeric ratio refers to C2:C3 regioisomers.

**Scheme 5:**
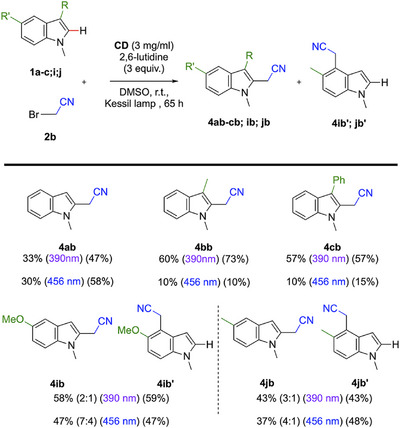
Substrate scope for the photoreaction with bromoacetonitrile **2b** and indole derivatives, with different light sources. Values in brackets represent the conversion values of the indole substrates, calculated via ^1^H NMR of the crude. Unless otherwise noted the reaction conditions were: **1** (0.1 mmol), **2b** (0.15 mmol), 2,6‐lutidine (0.3 mmol), **CD** (3 mg/mL) were dissolved in 1 mL of DMSO, degassed with Ar for 20 minutes and left stirring under irradiation for the indicated time.

**Scheme 6:**
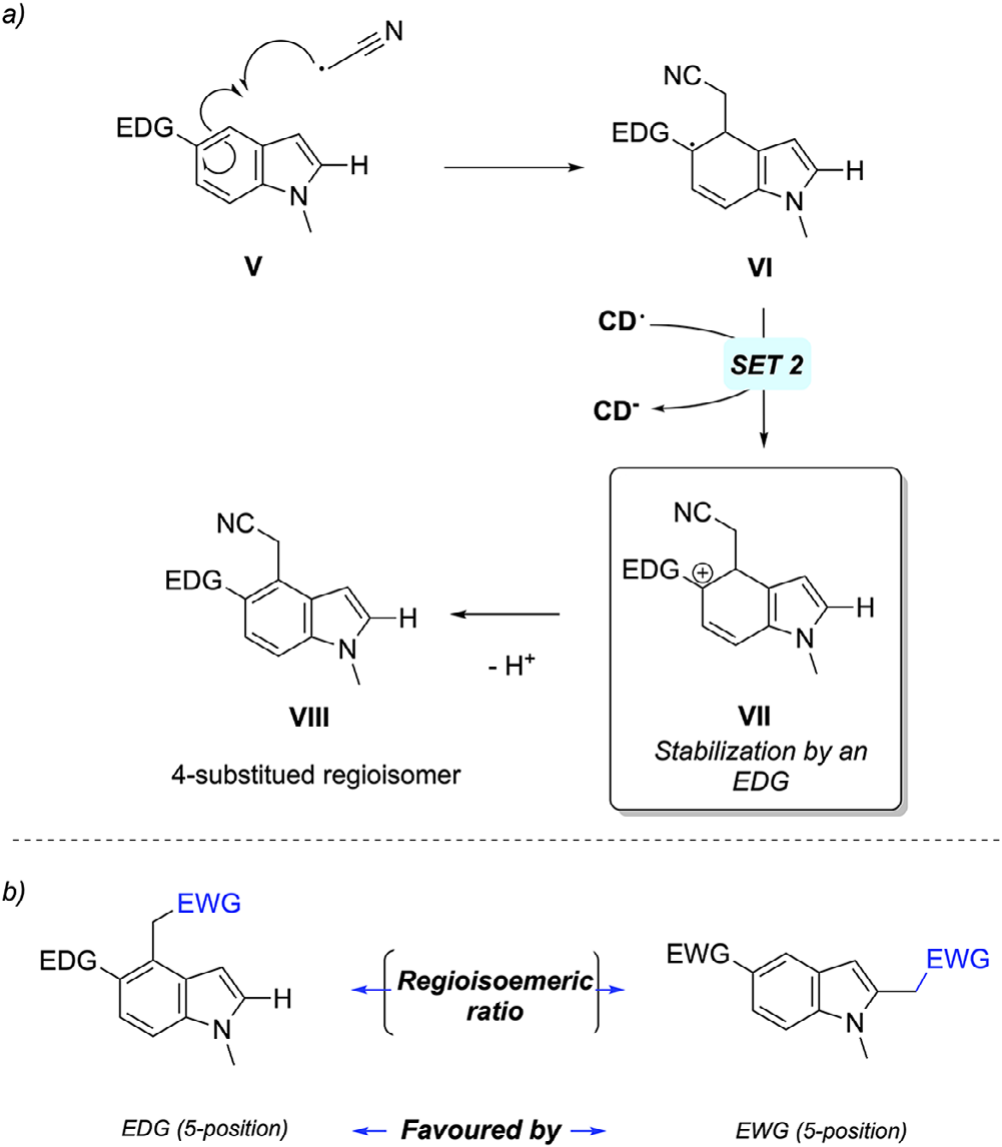
a) Explanation for the formation of C4 regioisomers. b) Parameters influencing the regioisomeric ratio between C2 and C4 substituted indoles formation.

With the optimized reaction conditions in hand, we addressed to heteroarene substrates (like indoles, furans, and pyrroles) using as electrophiles the radicals generated from diethyl bromomalonate **2a** and bromoacetonitrile **2b**. Initially, indoles scope **1a** to **1k** with reagent **2a** was studied (Scheme 4). First and foremost, different types of functional groups were tolerated by the reaction, including alkyl, phenyl, aldehyde, ester, ketones, methoxy, and halogens. 3‐substituted indoles with electron donating or stabilizing groups, such as **1b** and **1c**, presented high yields up to 88% (**3ba** and **3ca**). Reactions of 3‐substituted indoles with EWG groups showed lower yields from 29% to 32% (**3 da** to **3fa**), but as depicted before, making the related substrate react is still today a great challenge for C2 indole functionalization. Finally, different 5‐substituted indoles with both EWG and electron donating groups (EDG) were functionalized in average yield, from 26% to 57% (**3ga** to **3ka**), even showing that the reaction well tolerate the presence *N*‐Boc protecting group (**3ga**). The presence of EDG on C5 led to the formation of C4 substituted regioisomers (see **3 ha** and **3ia**), while the presence of EWG on C5 led to the formation of C3 substituted regioisomers (see **3ja** and **3ka**), along with the desired C2 functionalization. This issue of regioselectivity is studied and discussed below (Schemes 5 and  6). We were also able to obtain the functionalized product from the unprotected indole with a satisfactory yield (**3la**). This reaction was also performed with other haloalkanes, like bromoacetophenone **2c** and bromomalononitrile **2d** (Scheme 4). With **2c**, the radical was generated, as (PhCOCH_2_)_2_ was detected by NMR (Supporting Information). It was possible to draw the conclusion that the radical was not favorable to form the indole derivative. For **2d**, the reaction led to the brominated product on the C2 position (see Supporting Information). For all the tested substrates we calculated also the conversion values. These values were calculated based on the unreacted indole substrate. The results indicated that, for some cases (e.g., **3fa**, **3 ha**, **3ia**, **3la**), side‐product formation occurred, presumably ascribable to undesired radical quenching, although it was not possible to isolate these side products.

Then, we explored the scalability of the reaction up to a 1 mmol scale. The reaction yielded the corresponding product **3ba** with comparable good yield (73% yield), confirming the synthetic applicability of the developed methodology.

As suggested by the slope of the Stern–Volmer plots discussed above (Scheme 2a), the average yield for indoles functionalization with the reagent **2b** was less effective than with **2a**. Moreover, also in this case, the use of 390 nm light irradiation led to higher yields, if compared with the 456 nm light. As previously mentioned, this is possibly due to a higher concentration of radicals, which presumably better matches the kinetic of the indole functionalization reaction. Product **4ab** to **4cb** were obtained in higher yield compared to the other, due to the presence of donating and stabilizing group on the C3 position under the UV light (Scheme 5), and overall, it was still possible to obtain products with yields ranging from 33% to 60%. The conversion values analysis indicated that, in these cases, no side‐product formation occurred, which could derive from the lower reactivity of bromoacetonitrile.

As it has been possible to notice in the scope for the bromomalonate, the reaction with 5‐substituted indoles introduced a lack of regioselectivity. This phenomenon, which seems to be much present with the use of bromoacetonitrile **2b**, was studied through the scope with this latter. First, the presence of a 4‐substituted regioisomer was proven using 2D NMR on the molecule **4jb’** (Supporting Information). Once this regioisomer was isolated, NMR spectra were used to determine the ratio between them without isolating the 4‐regioisomer in question for all the reaction with **3 h** and **3i**, including the formation of **3 ha** and **3ia**. The same method was used for the 3‐regioisomer formation, after the isolation of the regioisomer of **3ka** (SI). The reaction was also tested with the indole **1l**, which led to less efficiency than its methylated form **1a** (product **3la**). Thanks to the mechanism that we detailed earlier (Scheme 2b), it was possible to draw a plausible mechanism to yield a 4‐regioisomer **VIII** (Scheme 6a). For electron rich indoles on the 5‐position, the intermediate radical **VI** and the intermediate cation **VII** could be stabilized by an EDG, favoring its formation through the reaction. In order to prove our statements, we managed to find that the donating property of a function was correlated to the regioisomeric ratio more balanced between the 2‐substituted product and the 4‐substituted one. Indeed, the reaction with the indole **4j** shows a higher regioisomeric ratio than the 5‐methoxyindole **4 h**. As a matter of fact, no regioisomer were obtained without 5‐substituted indoles, such as products **4ab** to **4ac**, which aligns with our hypothesis.

Following this discovery, we sought to understand how this ratio was influenced by the other reaction parameters unrelated to the substrates themselves. As highlighted in Scheme 5, the ratio was not significantly affected by the use of UV or blue light. However, by comparing the reactions with **1i** and **1j** with both **2a** and **2b**, it was possible to mak a correlation between the regioisomeric ratio and the redox potentials of both diethyl bromomalonate **2a** (*E*_red_  = −0.62 V vs. SCE in CH_3_CN)^[^ ^34^ ^]^ and bromoacetonitrile **2b** (*E*_red_  = −1,46 V vs. SCE in CH_3_CN).^[^ ^35^ ^]^ This also correlates with what has been demonstrated during the discussion on the mechanism thanks to the Stern–Volmer plots, which underline an easier formation of the diethyl bromomalonate reduction in presence of 2,6‐lutidine and **CD** (Scheme 3a). These considerations, summed up in Scheme 6b, can also be applied to the reaction with **2a**, as indoles **1 h** and **1i** led to the formation of 4‐substituted regioisomers, while indoles **1j** and **1k** led to the formation of 3‐substituted regioisomers, due to the presence of EWG on the position 5.

**Scheme 7:**
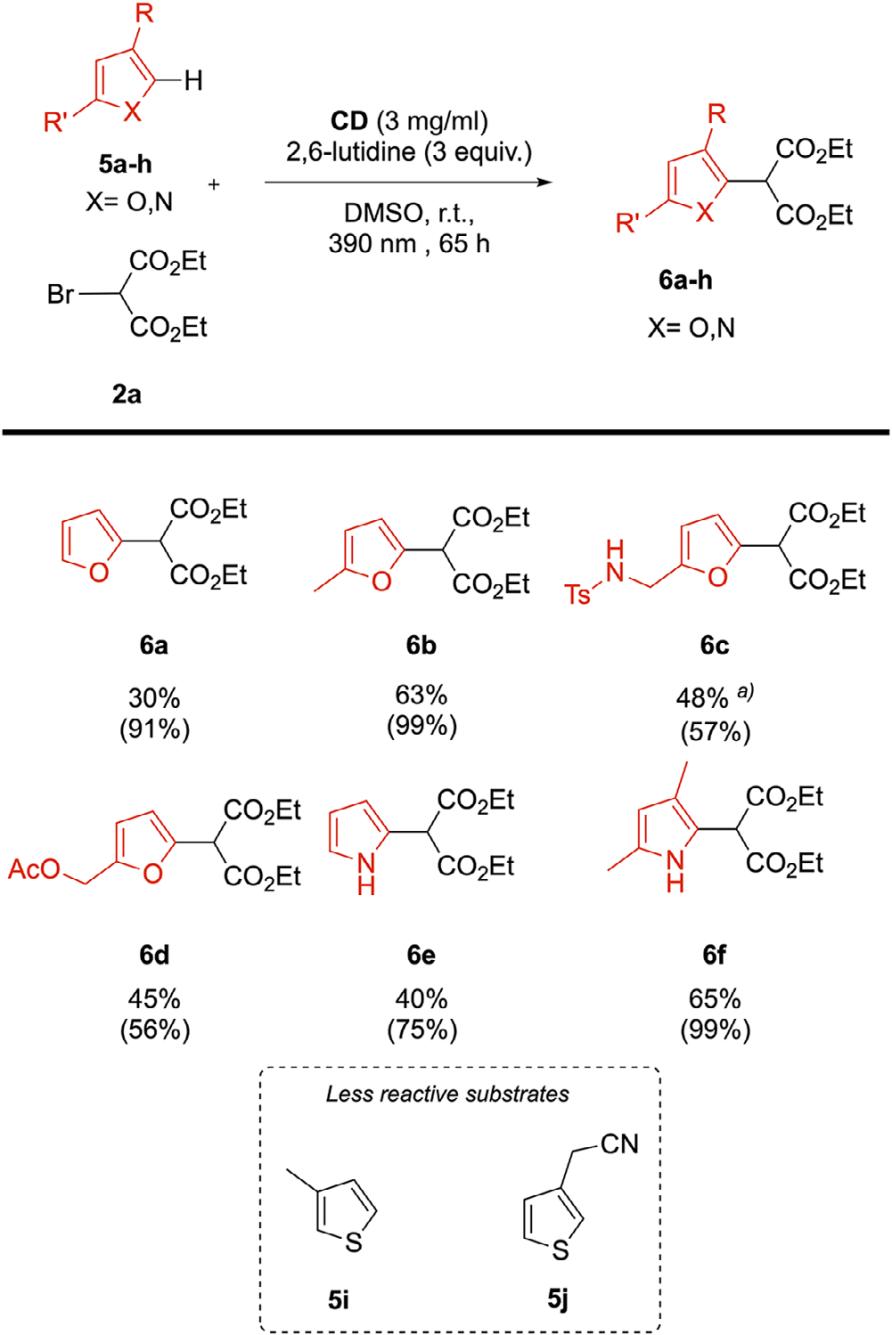
Reaction scope of the optimized photoreaction with diethyl bromomalonate **2a** on furane and pyrrolidine derivatives. Values in brackets represent the conversion values of the indole substrates, calculated via ^1^H NMR of the crude. Unless otherwise noted the reaction conditions: **5** (0.1 mmol), **2a** (0.15 mmol), 2,6‐lutidine (0.3 mmol), **CD** (3 mg/mL) were dissolved in 1 mL of DMSO, degassed with Ar for 20 minutes and left stirring under irradiation for the indicated time. a) reaction performed using the blue lamp since at 390 nm the degradation of the substrate occurred.

After the successful functionalization of indoles with bromomalonate, we decided to change the substrate nature for other heteroarenes, as these types of chemicals were also reactive in presence of 2,6‐lutidine (Scheme 7).^[^ ^18^ ^]^ As expected, we successfully obtained new types of functionalized heteroarenes with diethylmalonate moieties such as furanes (**6a–6d**) and pyrroles (**6e–6 h**). The high conversion values for these reactions could be derived from the high volatility of the starting materials, which may have affected the conversion determination.

The reaction was favored by electron rich substrates, as shown by the comparison of the yields of **6a** and **6b**, and **6e** and **6f**. The protected amino furfuryl **6c** led to a 48% yield under the blue light, as the reaction under the UV lamp led to a partial destruction of the protecting group. The reaction has also shown a certain efficiency toward thiophene derivatives such as **5i** and **5j**, but due to the low yields (less than 20%), the products were not isolated.

**Figure 2:**
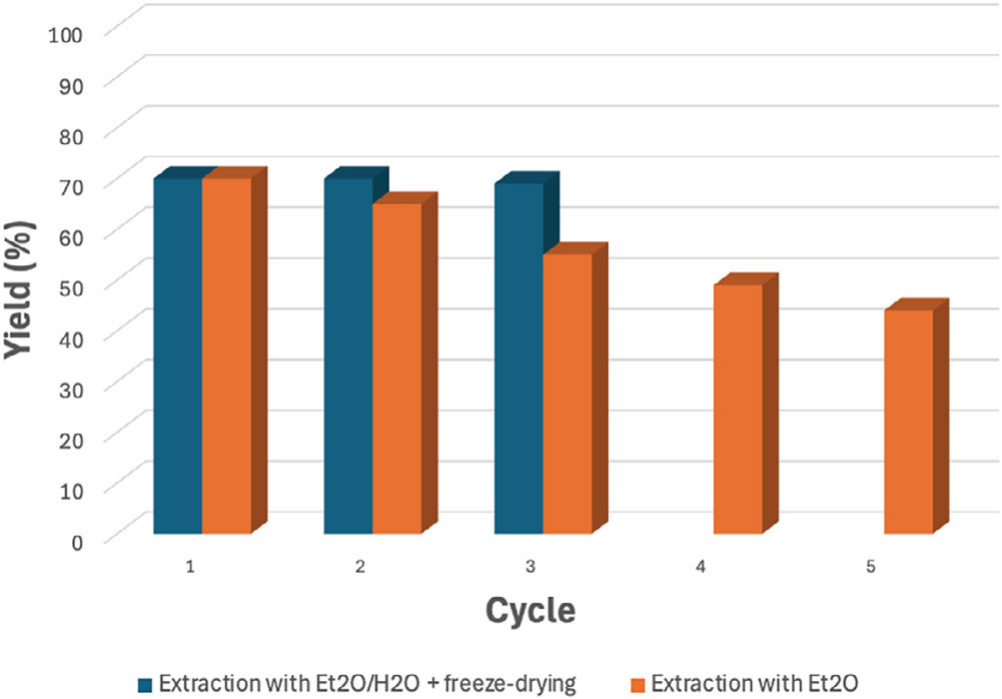
Catalyst recyclability experiments for the synthesis of model compound **3aa**. The reaction conditions were: **1** (0.2 mmol), **2** (0.3 mmol), 2,6‐lutidine (0.6 mmol), **CD** (3 mg/mL) were dissolved in 2 mL of DMSO, degassed with Ar for 20 minutes and left stirring under irradiation for 65 hours.

Following the extension of the scope, we investigated the potential recyclability of **CD** on a scaled‐up reaction (0.2 mmol). Initially, for our experimental design we decided, after the first reaction, to extract the organic compounds (product and reagents) with diethyl ether, relying on the partial immiscibility with DMSO. In this way, the **CD** stayed in the DMSO phase and could be directly used for the subsequent run by adding fresh reagents and degassing the solution. This set‐up proved to be operationally simple, leading to the formation of the desired product for up to five runs, with a slight decrease of yield (from 70% to 44%, Figure 2). We hypothesized that the yield drop was due to a partial loss of **CD** during the extraction process, since part of the DMSO passed in the ether phase, essentially diminishing at each run the amount of **CD** available in the DMSO phase. In fact, we proved that a lower amount of **CD** caused a decrease of yield to 52% by using 1 mg/mL of nanocatalyst (see Supporting Information). On this basis, we decided to run a different recyclability study, changing the work‐up step. After each reaction cycle, water was added to the reaction mixture and was then extracted with Et_2_O. Then, the water/DMSO mixture was freeze‐dried and subsequently dissolved in fresh DMSO for the next reaction cycle. In this case, no yield decrease was observed up to three reaction runs, proving excellent catalyst recyclability.

The presence of water, despite a longer work‐up, guaranteed a better phase separation, impeding nanocatalyst loss in the organic phase.

The fluorescence and XPS analyses of the used CDs did not show substantial differences with the freshly prepared ones, confirming the high photostability of the designed nanoparticles (see Supporting Information).

## 3 Conclusion

In conclusion, we reported in this work an extensive study on the photochemical and photocatalytic properties of carbon nanodots in the generation of alkyl radicals, which were employed in the functionalization of indoles and other heteroarenes. A detailed mechanistic investigation led us to define the role of 2,6‐lutidine in the alkyl radical generation, via fluorescence quenching studies, and the quantum yield calculation allowed the definition of the mechanistic pathway, which involved a direct photocatalytic mechanism. Subsequently we were able to successfully apply our nano‐photocatalysts in the alkylation of variously substituted indoles, with good to excellent results (up to 88% yield), using either bromomalonate or bromoacetonitrile as alkylating agents. Moreover, the optimized methodology was applied to the functionalization of other heteroarenes, with comparable good results. Lastly, we successfully demonstrated the recyclability of the nanocatalysts, confirming the promising applicability of the nanosystems as heterogeneous and sustainable photocatalysts.

## 4 Experimental Section

### General procedure for the nano‐photocatalytic alkylation of heterocycles

**CD** (3.0 mg) are added to a 4 mL oven‐dried vial equipped with a stirring bar, followed by dimethyl sulfoxide (1.0 mL), the indole (0.10 mmol, 1.0 equiv), the bromide (0.15 mmol, 1.5 equiv) and 2,6‐lutidine (35 µL, 0.3 mmol, 3.0 equiv). Depending on the functionalization, diethyl bromomalonate (26 µL) or bromo acetonitrile (10,5 µL) were used. The vial is closed with a rubber cap and the reaction mixture is degassed by bubbling Ar with a syringe for 20 minutes. The reaction is set at a distance of 5 cm from a 390 nm Kessil lamp equipped with a cooling fan. After 65 hours the mixture was diluted with brine, extracted with Et_2_O (3×20 mL), and the reunited organic layers were washed with water (20 mL), brine (20 mL), dried over anhydrous Na_2_SO_4_ and reduced in volume through rotary evaporation. The residue was purified with silica gel column chromatography using as eluant mixtures of n‐pentane and Et_2_O for the functionalization with diethyl bromomalonate and mixtures of n‐hexane and EtOAc for the alkylation with bromo acetonitrile. The NMR yields were determined using 1,3,5‐trimethoxybenzene (16.82 mg, 0.10 mmol, 1.0 equiv) for the alkylation with diethyl bromomalonate or mesitylene (14 µL, 0.1 mmol, 1.0 equiv.) for the functionalization with bromo acetonitrile.

## Supporting Information

The authors have cited additional references within the Supporting Information.^[^ ^15^, ^17^, ^18^, ^22^, ^36^ ^]^

CDs preparation, photochemical characterization, actinometry, control experiments, and screening, general procedures, and all analytical data.

## Conflict of Interest

The authors declare no conflict of interest.

## Supporting information



Supporting Information

## Data Availability

The data that support the findings of this study are available in the supplementary material of this article.
